# Outcomes of Anterior Chamber, Sulcus, and Pars Plana Glaucoma Drainage Device Placement in Glaucoma Patients

**DOI:** 10.1155/2022/5947992

**Published:** 2022-07-20

**Authors:** Sandy Samuel, Enchi K. Chang, Sanchay Gupta, Marika Chachanidze, Cameron E. Neeson, John B. Miller, Ta Chen Chang, David A. Solá-Del Valle

**Affiliations:** ^1^Massachusetts Eye and Ear, Harvard Medical School, Boston, MA, USA; ^2^Massachusetts Eye and Ear, Boston, MA, USA; ^3^Bascom Palmer Eye Institute, University of Miami Miller School of Medicine, Miami, FL, USA

## Abstract

**Purpose:**

To assess outcomes of anterior chamber (AC), sulcus, and pars plana (PP) glaucoma drainage device (GDD) placement in glaucoma patients. *Patients and Methods*. Retrospective evaluation of glaucoma patients who underwent GDD insertion in the AC, sulcus, or PP at Massachusetts Eye and Ear between November 2016 and May 2021. Patients who received AC, sulcus, and pars plana tubes were selected using simple random sampling, and the first 40 patients meeting inclusion criteria were analyzed. Main outcome measures were cumulative success probabilities from Kaplan-Meier (KM) analyses, intraocular pressure (IOP), medication burden, and complication rates.

**Results:**

The PP group had a larger proportion of Ahmed GDDs and was younger on average with less severe glaucoma compared to patients with AC or sulcus tubes. The PP group had a higher proportion of mixed-mechanism glaucoma and lower proportion of primary open-angle glaucoma. With success defined as IOP reduction ≥20% and 5 < IOP ≤ 21 mm Hg, the Kaplan-Meier cumulative success probabilities for all three GDD locations were not significantly different. No significant differences were found in complication rates between all groups after 3 months. Patients with PP GDD had significantly lower medication burden than those with AC or sulcus GDDs up to 1.5 years postoperatively (1.7 ± 1.1, 3.0 ± 1.4, and 2.8 ± 1.2 for PP, AC, and sulcus, respectively; *P*=0.017).

**Conclusion:**

PP GDDs may be more effective in lowering medication burden than AC or sulcus tubes without compromising long-term safety.

## 1. Introduction

Glaucoma drainage devices (GDDs) have been extensively utilized as a surgical option in the treatment of refractory glaucoma. A review of Medicare data revealed that 22,862 trabeculectomies (13.1% of all glaucoma surgeries) and 19,991 GDD insertions (11.4% of all glaucoma surgeries) were performed in the United States in 2017, indicating that GDDs were implanted almost as frequently as trabeculectomies were performed [1]. Depending on the patient's ocular history and comorbidities, these devices can be inserted into the anterior chamber (AC), the sulcus, or the pars plana (PP). Traditionally, GDDs are placed in the AC. This approach, however, may lead to tube-cornea touch or endothelial decompensation [2]. Hence, in patients with concurrent corneal disease, pars plana tube placements have been performed in lieu of AC insertion to prevent exacerbation of existing pathology [2]. Tube insertion through the ciliary sulcus is also a possible alternative in pseudophakic/aphakic patients or in patients with peripheral anterior synechiae [3]. Further description of the three surgical approaches is detailed in the Patients and Methods section.

Each of these placement approaches is associated with a unique set of disadvantages. Tube insertion into the anterior chamber can increase the risk for persistent corneal edema, depending on the tube length and specific location in the anterior chamber [4]. In previous studies, the frequency of corneal complications after GDD implantation into the anterior chamber ranged between 7% and 27% [5–8]. As a result, tube placement in the sulcus may be preferable as it may reduce endothelial cell loss in pseudophakic patients [9]. On the other hand, sulcus placement is likely safest when performed in pseudophakic or aphakic eyes. Although the vitreous cortex is greatly involved in tube occlusion, there are other components of the vitreous that can theoretically clog the tube [10, 11].

A three-way comparison of the safety and efficacy of GDD insertion in the AC, sulcus, and PP has not been reported previously to our knowledge. However, comparisons of AC and sulcus tubes, or AC and PP tubes, have been documented in the literature. For example, a study comparing the percent of IOP reduction between AC and sulcus GDDs noted that the percent reduction was significantly higher in the sulcus cohort [3]. Meanwhile, Tojo et al. [10] compared the safety and efficacy of GDD implantation in AC versus PP, demonstrating comparable IOP reduction in both groups. This study aims to examine differences in surgical outcomes and complication rates across these different GDD placement approaches.

## 2. Patients and Methods

### 2.1. Study Design

This is a retrospective cohort study of glaucoma patients who underwent GDD insertion in the anterior chamber, sulcus, or pars plana at Massachusetts Eye and Ear. Approval was obtained from the Mass General Brigham Institutional Review Board, and data collection abided by the Declaration of Helsinki and the Health and Portability and Accountability Act. Medical records of patients who underwent GDD insertion between November 2016 and May 2021 were identified and reviewed. Patients who received AC, sulcus, or PP tubes were randomly drawn using a simple random sampling approach, and the first 40 eyes in each group meeting the inclusion criteria were selected for analysis. GDD insertion was performed by 9 different providers, and PPV was performed by 8 different providers. Inclusion criteria were as follows: (1) GDD implantation in the AC, sulcus, or PP and (2) at least 3 months of follow-up without additional procedures. For patients who received procedures in both eyes, the first eye was included in our study. Patients below 18 years of age were excluded.

Preoperative, perioperative, and postoperative data collected included IOP, number of glaucoma medications, visual acuity (VA), additional IOP-lowering procedures, type of GDD, GDD tube location, and the presence of complications, including anterior chamber inflammation, hypotony, corneal edema, and cystoid macular edema (CME). IOP was measured with Goldmann applanation tonometry. Preoperative IOP, number of glaucoma medications, and VA were recorded as an average of the measurements from the two visits prior to the procedure. Additional preoperative characteristics included patient age, sex, glaucoma diagnosis and stage, and prior ocular or laser procedures. Postoperative data were obtained at the following time points after surgery: 1 day (POD1), 2 weeks (POW2), 6 weeks (POW6), 3 months (POM3), 6 months (POM6), 1 year (POY1), 1.5 years (POY1.5), and 2 years (POY2). Glaucoma stages were determined using optical coherence tomography and Humphrey visual field findings [12].

### 2.2. Surgical Procedure

A peribulbar block was placed by anesthesia. The patient's operative eye and ocular adnexa were sterilized with 5% Betadine solution and draped in a sterile fashion. A lid speculum was placed in the operative eye. A 7–0 polyglactin traction suture was placed, and a peritomy was performed. The sub-Tenon's space was accessed and dissected posteriorly.

All patients underwent either Ahmed (New World Medical, Rancho Cucamonga, CA, USA) or Baerveldt (Johnson and Johnson Vision, Santa Ana, CA, USA) GDD implant placement. The type of glaucoma implant and insertion location were at the surgeon's discretion. For Ahmed placement, the Ahmed GDD was brought onto the field and primed. The Ahmed implant was then fixed to the sclera with 2 interrupted 8-0 Nylon sutures. In general, viscoelastic material was left in the eye at the end of the case to prevent early postoperative hypotony. For Baerveldt placement, the recti muscles were identified. Next, the Baerveldt glaucoma drainage implant was brought onto the field and tested for patency. The wings of the plate were carefully placed under the previously identified rectus muscles. The GDD was then secured to the sclera using 2 interrupted 8-0 Nylon sutures. The tube was then ligated with a 7-0 polyglactin suture, and irrigation was attempted to confirm complete tube occlusion. Fenestrations were made at the surgeon's discretion.

### 2.3. Anterior Chamber Implantation

A superotemporal conjunctival peritomy was created, and the sub-Tenon's space was accessed and dissected posteriorly. A channel was created using a 23-gauge needle approximately 1.5–2 mm from the limbus. The tube was then trimmed to the appropriate length, cut in a bevel-up manner, and then inserted into the anterior chamber. The tube was covered with a cadaveric corneal patch graft and sutured to the sclera. Interrupted and running 8-0 polyglactin sutures were used to secure the overlying Tenon's capsule and conjunctiva at the limbus. 19 (48%) Ahmed glaucoma implants and 21 (42%) Baerveldt implants were used in this cohort.

### 2.4. Sulcus Implantation

For sulcus implantation, a paracentesis was created using a 75-blade through which viscoelastic material was injected behind the iris into the sulcus space. A channel was created using a 23-gauge needle approximately 2.5 mm from the limbus. The tube was then trimmed to the appropriate length, cut in a bevel-down manner, and then inserted into the sulcus. The tube was covered with a cadaveric corneal patch graft and sutured to the sclera. Viscoelastic material was removed from the eye for Baerveldt implants and was left in the eye for Ahmed implants. Interrupted and running 8-0 polyglactin sutures were used to secure the overlying Tenon's capsule and conjunctiva at the limbus. 3 (9%) Ahmed glaucoma implants and 37 (91%) Baerveldt implants were used in this cohort. Further details of this surgical technique can be found in a video of a similar surgery performed by one of the providers in this study [13].

### 2.5. Pars Plana Implantation

For the vitrectomy, trocars were used to place cannulas in the inferotemporal, superotemporal, and superonasal quadrants through the pars plana in a beveled fashion. A 4 mm infusion cannula was placed through the inferonasal cannula, and the infusion was confirmed in the vitreous cavity prior to turning it on. At this time, a standard three-port pars plana vitrectomy was performed. Two sclerotomies were closed using interrupted 7-0 polyglactin sutures, and the conjunctiva was closed in conjunction with the sclerotomy closure. The tube was cut at the appropriate length in a bevel-down manner. The remaining trocar was removed, and viscoelastic material was injected into the pars plana if an Ahmed GDD was being placed. Then either the tube was inserted into the pars plana approximately 4 mm from the limbus through the existing sclerotomy, or the existing sclerotomy was sutured closed and a new sclerotomy was created for the tube. The tube was covered with a cadaveric corneal patch graft and sutured to the sclera. Interrupted and running 8-0 polyglactin sutures were used to secure the overlying Tenon's capsule and conjunctiva at the limbus. 36 (90%) Ahmed glaucoma implants and 4 (10%) Baerveldt implants were used in this cohort. Further details of this surgical technique can be found in a video of a similar surgery performed by one of the providers in this study [14].

### 2.6. Outcome Measures

Primary outcome measures at each postoperative visit included cumulative success probabilities from Kaplan-Meier (KM) analyses, IOP reduction, glaucoma medication burden, and complications. For survival analysis, the success criteria were defined as follows: IOP reduction ≥20% from preoperative levels with 5 mm Hg < IOP ≤21 mm Hg. A failure was recorded if patients did not meet the specified success criteria or developed no light perception vision on two consecutive follow-up visits after 3 months, with the latter of the two dates defined as the failure date. Patients who required an additional IOP-lowering procedure were considered as failures on the date of the second surgery. Secondary outcome measures in this study were VA and complication rates. Complications up to 3 months after the surgery were considered “early” complications, and complications after 3 months were termed “late” complications.

### 2.7. Statistical Analysis

R (version 4.0.2) was used for all statistical analyses. Statistical significance was established at a *P* value of 0.05. Survival analysis was conducted by constructing Kaplan-Meier curves to determine success probabilities, and Cox proportional hazard regression analyses were conducted to obtain hazard ratios for preoperative characteristics. Average values for postoperative IOP, number of medications, and VA were calculated along with standard deviations (SD). These values were plotted online graphs, and the standard errors of the means were represented using error bars. Preoperative and postoperative values were compared using Wilcoxon paired signed-rank tests. Snellen visual acuities were converted to their equivalent logarithm of minimum angle of resolution (LogMAR) for comparisons. Count fingers and hand motion vision were represented with LogMAR values of 2 and 3, respectively. LogMAR equivalents were not calculated for patients with light perception and no light perception vision. These patients were also not included in mean calculations and paired Wilcoxon testing. Overall, statistical analysis was conducted as previously described in a work by Chang et al. [15].

## 3. Results

Patient demographics and preoperative baseline data are provided in Tables 1 and 2. A total of 120 eyes of 120 glaucoma patients were included in this study. Mean age was 69.8 ± 15.1 years with a range of 18–92 years. 53.3% of patients in the study were female. The vast majority of patients had severe stage glaucoma (74.2%). With respect to glaucoma type, the diagnoses with the highest prevalence in the study were primary open-angle glaucoma (45.0%), followed by mixed-mechanism (30.0%), and pseudoexfoliative glaucoma (15.8%). Mean preoperative IOP was 22.3 ± 7.4 mm Hg and ranged from 9 to 49 mm Hg. Mean preoperative medication burden was 4.1 ± 1.1 and ranged from 1 to 6 medications. The average follow-up period was 16.4 ± 10.1 months.

Information about the type of GDD (AGI versus BGI) and the location of GDD insertion (anterior chamber, sulcus, or pars plana) is presented in Table 2. Overall, 58 patients (48.3%) underwent AGI insertion, and 62 patients (51.7%) underwent BGI insertion. Patients with tube insertion in the PP were younger and had less severe glaucoma than patients with tube insertion in the AC or sulcus. The PP tube group also had a higher proportion of patients with mixed-mechanism glaucoma and a lower proportion of primary open-angle glaucoma patients compared with AC or sulcus tubes. With regard to lens status, a larger proportion of pseudophakic patients were in the AC and PP tube groups and phakic patients in the sulcus tube group. The sulcus tube group had a lower starting IOP than the AC and PP tube groups. All phakic patients in the sulcus group received concurrent phacoemulsification, and all patients with PP tube insertion received a concurrent pars plana vitrectomy.

**Figure 1:**
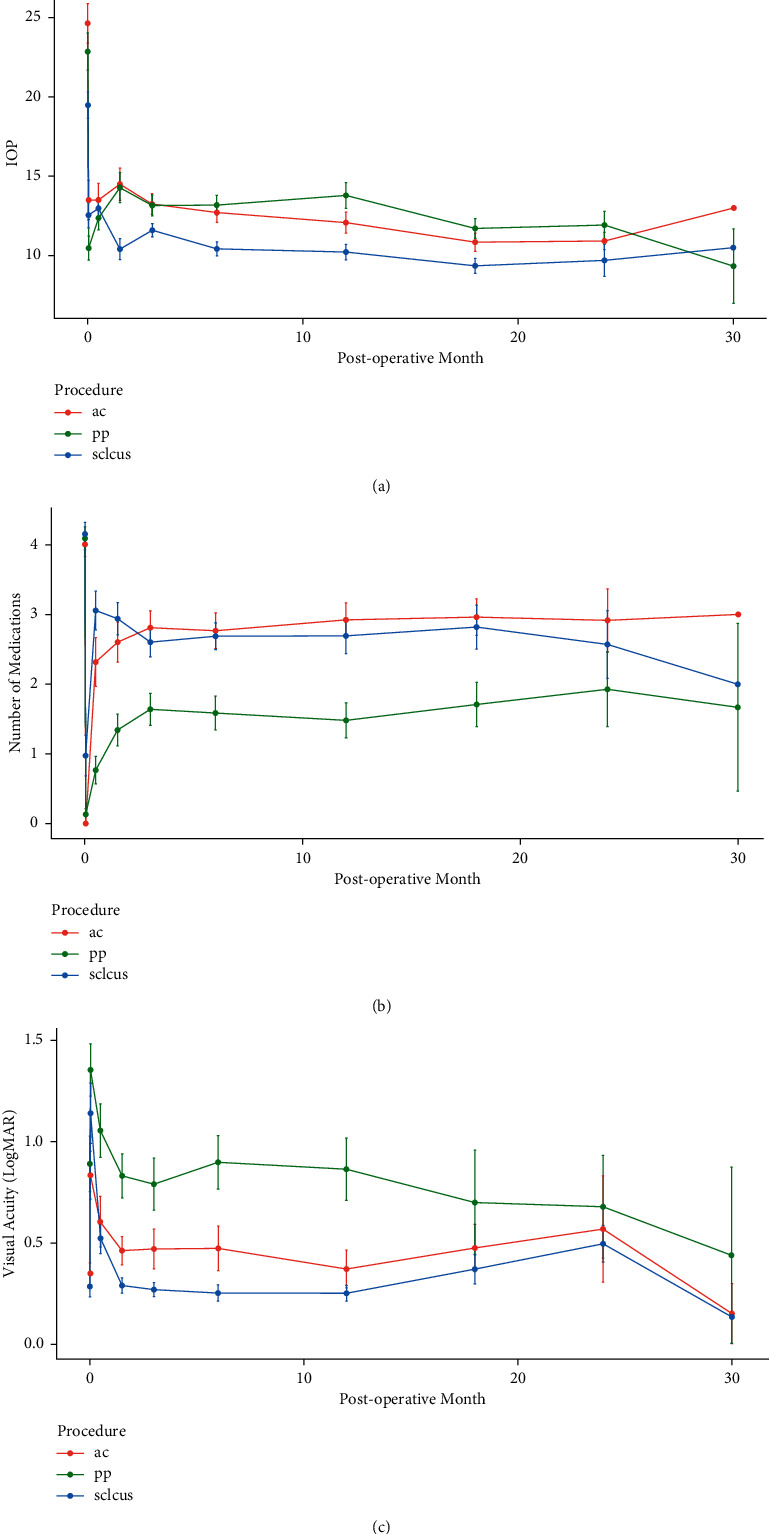
Line graphs of average values of postoperative (a) intraocular pressure, (b) number of medications, and (c) visual acuity over time. Error bars denote standard error of the mean.

Table 3 summarizes the mean preoperative IOP and the IOP at each of the postoperative time points for the groups. Postoperative medication burden and VA outcomes across groups are summarized in Table 4 and Table 5, respectively. Line graphs of these postoperative outcomes are shown in Figure 1. Postoperative IOP was significantly decreased from preoperative baseline at all follow-up time points across groups. The sulcus group had lower IOP than the AC and PP groups at baseline, and this trend largely persisted up to 1.5 years (9.4 ± 1.8, 11.7 ± 2.1, and 10.8 ± 3.0 mmHg for sulcus, PP, and AC, respectively; *P* = 0.034). Patients with a PP GDD had a significantly lower medication burden at all time points compared to patients with AC or sulcus insertion up to 1.5 years postoperatively (1.7 ± 1.1, 3.0 ± 1.4, and 2.8 ± 1.2 for PP, AC, and sulcus, respectively; *P* = 0.017). The change in VA from baseline values was not significantly different between groups at all time points.

**Figure 2:**
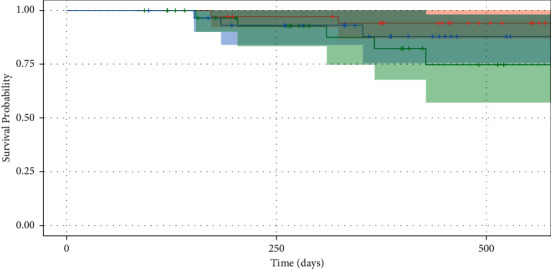
Kaplan-Meier survival curves of glaucoma drainage device placement in the anterior chamber (red), sulcus (blue), and pars plana (green). Success criteria were defined as the following: postoperative intraocular pressure (IOP) reduction ≥20% with 5<IOP.

Cumulative success probabilities based on Kaplan-Meier analysis are shown in Table 6, with the corresponding Kaplan-Meier curve depicted in Figure 2. The cumulative success probabilities for all three groups were not significantly different (*P*=0.2). Hazard ratios along with 95% confidence intervals from Cox proportional hazard regression analyses are reported in Table 7. The hazard ratios for a sulcus tube and a PP tube compared with an AC tube were both not significant. The hazard ratio for baseline IOP was 0.594 (*P* < 0.001). Additionally, the hazard ratios for sex, age, glaucoma stage, family history of glaucoma, baseline medication burden, history of cyclophotocoagulation laser procedures, and type of glaucoma tube inserted were not statistically significant.

Postoperative complication rates for the three groups are summarized in Table 8. No significant differences were noted between any groups after 3 months postoperatively. In the AC group, AC inflammation resolved after 3 months and reappeared at 10 months in a single eye. Corneal edema was present in one eye at all follow-up time points and was noted at 1.5 years in two eyes. One AC patient developed corneal edema at 2 years after undergoing micropulse transscleral cyclophotocoagulation. In the PP group, AC inflammation was also found at 1 year in a single patient who developed bleeding from neovascular glaucoma. Corneal edema was present in 4 patients after 3 months, resolving at 6 months for two eyes, and 1.5 years for one eye while persisting through the last follow-up visit in one eye. CME developed postoperatively in 5 eyes in the AC group, of which one resolved spontaneously, and one was noted after a subsequent procedure. In the sulcus group, 6 eyes developed CME postoperatively with one eye experiencing spontaneous resolution. In the PP group, CME developed postoperatively in 8 eyes and resolved spontaneously in 3 eyes. Two patients in the PP group required repeat PPV prior to 3 months postoperatively for vitreous or blood clogging the tube.

At the last follow-up visit, VA was unchanged or improved from preoperative levels in 21 (52.5%), 23 (57.5%), and 28 (70.0%) eyes in the AC, sulcus, and PP groups, respectively. VA decreased by more than line in 6 (15.0%), 3 (7.5%), and 7 (17.5%) eyes in the AC, sulcus, and PP groups, respectively. Causes for the decrease in VA in these patients are summarized in Table 9.

## 4. Discussion

To the best of our knowledge, there is no literature assessing the direct three-way comparison of the safety and efficacy of GDD insertion in the AC, sulcus, and PP prior to this study. There was no significant difference in success rate between the AC, sulcus, and PP groups, where success was defined as IOP reduction ≥20% from preoperative levels with 5 mm Hg < IOP ≤ 21 mm Hg. Furthermore, no significant differences were found in complication rates between all groups after 3 months. However, the PP group's reduction in medication burden was significantly greater than that in the AC and sulcus groups (up to 1.5 years postoperatively).

Of note, there were a number of differences in baseline characteristics between groups which must be taken into account. Namely, all groups varied in their age, glaucoma stage, glaucoma type, lens status, prior laser history, prior surgical history, VA, IOP, and type of GDD implant. Previous studies have suggested that management of glaucoma in younger patients is typically more aggressive than in older eyes due to a larger cumulative impact of elevated IOP on the optic nerve [16, 17]. It is therefore notable that medication burden was significantly lower in the pars plana group, given that this cohort was relatively younger than the AC and sulcus cohorts and we expected medication burden to be higher based on age. With respect to glaucoma stage, the authors have recommended using a lower target IOP and therefore more aggressive medical/surgical management in eyes with more severe disease [17]. However, the target IOP concept is variably applied across providers and the rate at which glaucoma can progress at a given IOP level is unpredictable, thereby limiting reliance on this approach [18]. Furthermore, through Cox proportional hazard regression analyses, we found that variables such as age, glaucoma stage, and implant type did not affect the probability of success in our cohort. With regard to differences in lens status between the three groups, there is currently limited literature on the association between lens status and the success of tube implants. However, the Tube versus Trabeculectomy (TVT) Study suggested that lens status was not associated with treatment failure [19]. In addition, ﻿given that a larger proportion of patients with PP tubes had a prior cyclophotocoagulation (CPC) procedure, it is possible that the effects of prior CPC could result in a greater IOP reduction in this group; however, based on hazard ratios, we found that having a prior CPC did not affect the probability of success in our cohort.

For our success criteria as defined in our methods, there were no significant differences between the cumulative success probabilities between tube locations. The hazard ratio for baseline IOP suggests that each incremental increase in baseline IOP results in a 40.6% decrease in the risk of failure to achieve our specified success criteria. Thus, a higher preoperative IOP was associated with a higher success rate of achieving at least a 20% IOP reduction, similar to the findings from prior studies [20–24]. Furthermore, previous studies comparing Ahmed and Baerveldt GDDs outcomes have demonstrated lower failure rates and lower postoperative medication burden with Baerveldt tubes [25, 26]. Thus, given that the majority of GDDs implanted in the pars plana in our study were Ahmed GDDs, it is possible that a greater reduction in IOP would have been observed if Baerveldt GDDs were more predominant in this group.

Postoperative IOP was significantly decreased from preoperative baseline IOP at all follow-up time points, with mean reductions from 24.6 to 10.8 mm Hg in the AC, from 22.9 to 11.7 mm Hg in the PP, and from 19.5 to 9.4 mm Hg in the sulcus group at 1.5 years. The magnitude of this IOP reduction was found to be greater in the AC group (*P* = 0.035), although this is likely due to the fact that baseline IOP was higher in eyes with AC tubes. Baseline IOP has previously been shown to correlate with magnitude of IOP reduction, which would largely explain this effect [27]. It is difficult to compare these results directly to prior studies due to differences in study populations (glaucoma type, severity, and baseline IOP). Although studies conducting a three-way comparison across GDD insertion locations have not been performed previously, comparisons of AC and sulcus tubes, or AC and PP tubes, have been reported in the literature. Bayer et al., for instance, showed a last-visit mean IOP reduction from 36.9 mm Hg to 16.4 mm Hg after AC Ahmed valve implantation and reduction from 37.6 mm Hg to 14.4 mm Hg in the sulcus group, with an average follow-up time period of 27.2 months in the AC group and 30.2 months in the sulcus group [3]. While the difference in last-visit IOP between groups was not significant (*P* = 0.06), the authors found that the percentage of IOP reduction from baseline was significantly higher in the sulcus group (*P* = 0.03). In a study of 105 patients who underwent AGI or BGI implantation in either the AC or sulcus, Alobaida et al. [28] reported a comparable IOP decrease between groups at 16.5 months and 14.2 months, respectively (AC: 32.2 mm Hg to 18.1 mm Hg; sulcus: 31.5 mm Hg to 16.7 mm Hg). Meanwhile, Tojo et al. [10] compared safety and efficacy of BGI implantation in AC versus PP and showed a 2-year mean IOP reduction from 30.2 mm Hg to 12.0 mm Hg in the AC and from 32.4 mm Hg to 11.1 mm Hg in the PP group.

Additionally, we found significant reductions in medication burden from baseline levels across all groups postoperatively through POY1.5. Interestingly, this reduction was significantly more pronounced in the PP group relative to the AC and sulcus groups up to 1.5 years. However, significant differences in medication burden between tubes placed in the AC and sulcus, or AC and PP, have not been found previously to our knowledge. Tojo et al. [10] showed a similar trend in medication burden reduction, from 3.7 to 2.6 with AC insertion and from 3.5 to 1.9 with PP insertion at POY2, although the comparisons between groups were not significantly different. Two prior studies found no differences in medication burden preoperatively or at the last follow-up visit for tubes placed in the AC or sulcus, with average follow-up periods ranging from 2 to 3.5 years [0 3, 0 29]. Given the fact that there were no significant differences in preoperative medication burden or success rates across groups, our results suggest that patients who underwent GDD in the PP were able to achieve similar levels of IOP control with fewer glaucoma medications.

Interestingly, the long-term effects of PPV on IOP are controversial. PPV has traditionally been associated with increased risk of elevated IOP in the long term [29, 30]. However, in a study of 68 patients who underwent simple PPV, Yang et al. noted a decrease in mean postoperative IOP [31]. They postulated that PPV patients in prior studies also had other risk factors for IOP elevation, including severe diabetic retinopathy or combined PPV procedures like scleral buckling and tamponade with gas or silicone oil [31]. To complicate the discussion further, others have reported no long-term change after PPV, even when combined with phacoemulsification [32].

With respect to surgical complications, it has previously been shown that AC tubes are associated with higher rates of endothelial cell loss compared to sulcus tubes in pseudophakic patients as measured by specular microscopy [9, 33]. Such observations support the theory that sulcus or PP implantation of GDDs may minimize complications stemming from the proximity between the implant and the corneal endothelium. Alobaida et al. [28] reported hyphema in 17/60 patients who underwent GDD in AC versus only 3 instances of hyphema out of 45 patients who underwent sulcus tube placement (*P*=0.005). The authors also reported significantly higher severe or late-stage complications in the AC group, including implant exposure, corneal decompensation, endophthalmitis, poor vision, choroidal hemorrhage, and corneal edema (13/60 eyes in AC versus 2/45 eyes in sulcus; *P*=0.013) [28]. The length and specific positioning of the tube in the AC have previously been postulated to contribute to the risk of corneal endothelial damage and thus may potentially explain these discrepancies in complication rates [34]. Adding to the controversy, Qin et al. [35] found no significant differences in complication rates in a comparison of 57 AC versus 57 PP patients who underwent GDD insertion.

Our results suggest similar long-term complication rates across all GDD insertion locations. Although sulcus tubes were associated with higher short-term rates of AC inflammation and corneal edema in our study, these differences resolved after 3 months. Potential explanations for higher short-term postoperative edema and inflammation in sulcus patients include coupling of GDD insertion with a second procedure, phacoemulsification, and increased manipulation of the ciliary process during tube insertion. While we previously hypothesized that PP tubes would be associated with an increased risk of CME, there were no statistically significant differences in CME rates among tube locations. The change in VA from baseline values was not significantly different between groups at all time points. These findings may be reassuring to ophthalmologists who are facing a choice of where best to place the tube. A comparison of endothelial cell morphology in all groups would help further refine the hypothesis that tube placement in the sulcus or pars plana is safer for the cornea than traditional AC placement.

Some limitations of this study include its retrospective design, variability in baseline characteristics, the complex patient population at MEE, and lack of data at later time points for a number of patients. Future studies comparing cohorts with greater baseline similarity may be helpful in elucidating whether this is the case. Moreover, subgroup analysis by provider and by GDD type (Ahmed versus Baerveldt) was not conducted due to small sample sizes when stratifying by these variables. We thus could not evaluate the safety and efficacy of individual GDD types.

## 5. Conclusions

This is a comparative study that aims to explore the safety and efficacy of GDD insertion in the AC, sulcus, and PP. Our findings support the idea that tube implantation through the pars plana is likely a safe and effective alternative to implantation in the AC or sulcus. IOP reduction was statistically significant in all three cohorts with AC tubes resulting in the greatest magnitude of IOP reduction. PP tubes may potentially achieve a superior reduction in medication burden compared to AC or sulcus tubes. In addition, long-term complication rates, such as AC inflammation and corneal edema, likely do not differ between tube insertion locations.

While there are different indications for the surgical approaches discussed in this study, comparing their efficacy and safety profile can inform providers when formulating treatment plans and counselling patients. Further studies may be indicated to expand our sample size over a longer period of time, control for differences in baseline characteristics, and explore the efficacy of Ahmed and Baerveldt GDD placement independently across the three locations.

## Figures and Tables

**Table 1:** Demographic and ocular data^a^.

Parameters	Total	AC	*S*	PP	*P* value
*Demographics*					
Eyes	120	40	40	40	
Female sex, N (%)	64 (53.3)	23 (57.5)	22 (55.0)	19 (47.5)	0.719
Age (years)					<0.001^*∗*^
Mean ± SD	69.8 ± 15.1	73.9 ± 11.0	72.9 ± 11.7	62.6 ± 18.9	
Range	18–92	46–92	43–87	18–90	

*Glaucoma stage, N (%)*					<0.001^*∗*^
Mild	7 (5.8)	0 (0.0)	0 (0.0)	7 (17.5)	
Moderate	20 (16.7)	3 (7.5)	4 (10.0)	13 (32.5)	
Severe	89 (74.2)	36 (90.0)	36 (90.0)	17 (42.5)	
Indeterminate	4 (3.3)	1 (2.5)	0 (0.0)	3 (7.5)	

*Glaucoma type, N (%)*					<0.001^*∗*^
Aphakic	1 (0.8)	0 (0.0)	0 (0.0)	1 (2.5)	
Chronic angle closure	6 (5.0)	0 (0.0)	2 (5.0)	4 (10.0)	
Mixed-mechanism	36 (30.0)	5 (12.5)	9 (22.5)	22 (55.0)	
Neovascular	3 (2.5)	2 (5.0)	0 (0.0)	1 (2.5)	
Normal tension	1 (0.8)	1 (2.5)	0 (0.0)	0 (0.0)	
Primary open-angle	54 (45.0)	24 (60.0)	23 (57.5)	7 (17.5)	
Pseudoexfoliative	19 (15.8)	8 (20.0)	6 (15.0)	5 (12.5)	

*Mixed-mechanism type, N (%)*					<0.001^*∗*^
Traumatic	10 (8.3)	2 (5.0)	0 (0.0)	8 (20.0)	
CACG	15 (12.5)	2 (5.0)	5 (12.5)	8 (20.0)	
POAG	13 (10.8)	2 (5.0)	4 (10.0)	7 (17.5)	
CRVO	2 (1.7)	0 (0.0)	2 (5.0)	0 (0.0)	
DM	1 (0.8)	0 (0.0)	1 (2.5)	0 (0.0)	
Narrow angles	2 (1.7)	0 (0.0)	1 (2.5)	1 (2.5)	
Pigmentary	3 (2.5)	1 (2.5)	1 (2.5)	1 (2.5)	
JOAG	2 (1.7)	1 (2.5)	1 (2.5)	0 (0.0)	
Steroid response	9 (7.5)	2 (5.0)	0 (0.0)	7 (17.5)	
High myopia	1 (0.8)	1 (2.5)	0 (0.0)	0 (0.0)	
Postvitrectomy	2 (1.7)	1 (2.5)	1 (2.5)	0 (0.0)	
Uveitic	5 (4.2)	0 (0.0)	1 (2.5)	4 (10.0)	
NVG	2 (1.7)	0 (0.0)	1 (2.5)	1 (2.5)	
ICE syndrome	1 (0.8)	0 (0.0)	1 (2.5)	0 (0.0)	
Aniridia	4 (3.3)	0 (0.0)	0 (0.0)	4 (10.0)	

*Lens status, N (%)*					<0.001^*∗*^
Phakic	44 (36.7)	8 (20.0)	30 (75.0)	6 (15.0)	
Pseudophakic	71 (59.2)	32 (80.0)	10 (25.0)	29 (72.5)	
Aphakic	5 (4.2)	0 (0.0)	0 (0.0)	5 (12.5)	

*Prior glaucoma laser, N (%)*					<0.001^*∗*^
None	60 (50.0)	22 (55.0)	26 (65.0)	12 (30.0)	
ALT	3 (2.5)	1 (2.5)	0 (0.0)	2 (5.0)	
LPI	14 (11.7)	1 (2.5)	7 (17.5)	6 (15.0)	
LTP	4 (3.3)	1 (2.5)	0 (0.0)	3 (7.5)	
MPCPC/CWCPC	22 (18.3)	5 (12.5)	1 (2.5)	16 (40.0)	
SLT	20 (16.7)	9 (22.5)	8 (20.0)	3 (7.3)	
YAG	7 (5.8)	3 (7.5)	0 (0.0)	4 (10.0)	
PRP	2 (1.7)	0 (0.0)	0 (0.0)	2 (5.0)	
ECP	3 (2.5)	0 (0.0)	0 (0.0)	3 (7.5)	

*Prior glaucoma surgery, N (%)*					<0.001^*∗*^
None	40 (33.3)	8 (20.0)	26 (65.0)	6 (15.0)	
Trabeculectomy	20 (16.7)	10 (25.0)	5 (12.5)	5 (12.5)	
Tube shunt	5 (4.2)	3 (7.5)	0 (0.0)	2 (5.0)	
PEcK, iStent	2 (1.7)	1 (2.5)	0 (0.0)	1 (2.5)	
Other (PPV, DSEK, Phaco)	74 (61.7)	28 (70.0)	10 (25.0)	36 (90.0)	

AC = anterior chamber; *S* = sulcus; PP = pars plana; *N* = number of eyes; SD = standard deviation; CACG = chronic angle closure glaucoma; POAG = primary open-angle glaucoma; CRVO = central retinal vein occlusion; DM = diabetes mellitus; JOAG = juvenile open-angle glaucoma; NVG = neovascular glaucoma; ICE = iridocorneal endothelial; ALT = argon laser trabeculoplasty; LPI = laser peripheral iridotomy; LTP = laser trabeculoplasty; MPCPC = Micropulse cyclophotocoagulation; CWCPC = continuous wave cyclophotocoagulation; SLT = selective laser trabeculoplasty; YAG = YAG laser capsulotomy; PRP = pan-retinal photocoagulation; PEcK = phacoemulsification with endoscopic cyclophotocoagulation and Kahook dual blade; PPV = pars plana vitrectomy; DSEK = Descemet's stripping endothelial keratoplasty; Phaco = phacoemulsification. ^a^Baseline demographic and ocular data across the 3 treatment groups were compared using a Fisher test.

**Table 2:** Preoperative and surgical data^a^.

Parameters	Total	AC	*S*	PP	*P* value
*Preoperative baseline*					
IOP (mmHg)					0.006^*∗*^
Mean ± SD	22.3 ± 7.4	24.6 ± 8.4	19.5 ± 5.3	22.9 ± 7.4	
Range	9–49	9–49	11–42	10.5–42.5	
Visual acuity					<0.001^*∗*^
Range	LP–20/20	LP–20/20	20/800–20/20	LP–20/20	
# of glaucoma medications					0.827
Mean ± SD	4.1 ± 1.1	4.0 ± 1.1	4.2 ± 1.1	4.1 ± 1.0	
Range	1–6	1–6	2–6	2–6	

*Type of surgery*					<0.001^*∗*^
AGI	19 (15.8)	17 (42.5)	2 (5.0)		
AGI/Phaco	3 (2.5)	2 (5.0)	1 (2.5)		
BGI	27 (22.5)	18 (45.0)	9 (22.5)		
BGI/Phaco	31 (25.8)	3 (7.5)	28 (70.0)		
AGI/PPV	31 (25.8)			31 (77.5)	
AGI/PPV/Phaco	5 (4.2)			5 (12.5)	
BGI/PPV	4 (3.3)			4 (10.0)	

*BGI fenestration* ^b^					
Yes	49 (79.0)	16 (76.2)	31 (83.8)	2 (50.0)	
No	13 (21.0)	5 (23.8)	6 (16.2)	2 (50.0)	

*Indications for sulcus placement, N (%)*					
Concurrent cataract surgery		30 (75.0)			
Pseudophakia		8 (20.0)			
Corneal transplant		3 (7.5)			

*Indications for PP placement, N (%)*					
Corneal transplant		15 (37.5)			
ACIOL		8 (20.0)			
SIOL		4 (10.0)			
Aphakia		4 (10.0)			
PPV needed for other indications		4 (10.0)			
PCIOL scleral fixated		2 (5.0)			
Shallow AC		1 (2.5)			
Fuchs dystrophy		1 (2.5)			
Presence of GDD in AC		1 (2.5)			

*Concurrent procedures in PP group*					
Removal of capsular remnants				8 (20.0)	
PRP				6 (15.0)	
Epiretinal membrane peel				6 (15.0)	
Posterior capsulotomy				1 (2.5)	
Intravitreal injection				1 (2.5)	
Fluid air exchange				1 (2.5)	
IOL repositioning/removal				2 (5.0)	

AC = anterior chamber; *S* = sulcus; PP = pars plana; IOP = intraocular pressure; mmHg = millimeters of mercury; SD = standard deviation; AGI = Ahmed glaucoma implant; BGI = Baerveldt glaucoma implant; PPV = pars plana vitrectomy; Phaco = phacoemulsification; *N* = number of eyes; ACIOL = anterior chamber intraocular lens; SIOL = sulcus intraocular lens; PCIOL = posterior chamber intraocular lens; GDD = glaucoma drainage device; PRP = pan-retinal photocoagulation; IOL = intraocular lens. ^a^Preoperative and surgical data across the 3 treatment groups were compared using a Fisher test. ^b^Percentages were calculated as a proportion of eyes in each group that underwent BGI.

**Table 3:** IOP outcomes data^a^.

	IOP (mmHg)			*P* value			
	*AC*	*S*	*PP*	*All groups*	*AC* ∼ *S*	*S* ∼ *PP*	*AC* ∼ *PP*
*Preoperative*							
*N*	40	40	40				
Mean (SD)	24.6 (8.4)	19.5 (5.3)	22.9 (7.4)	0.006^*∗*^	<0.001^*∗*^	0.018^*∗*^	0.148

*1 day*							
*N*	40	40	39				
Mean (SD)	13.5 (7.7)	12.5 (5.1)	10.5 (4.8)	0.107			
Decrease from baseline (SD)	11.1 (13.8)	6.9 (7.2)	12.1 (8.4)	0.014^*∗*^	0.033^*∗*^	0.002^*∗*^	0.146
*P* value compared to baseline	<0.001^*∗*^	<0.001^*∗*^	<0.001^*∗*^				

*2 weeks*							
*N*	38	37	39				
Mean (SD)	13.5 (6.3)	13.0 (4.3)	12.4 (4.8)	0.870			
Decrease from baseline (SD)	10.5 (10.6)	6.4 (6.7)	10.6 (9.8)	0.037^*∗*^	0.021^*∗*^	0.009^*∗*^	0.364
*P* value compared to baseline	<0.001^*∗*^	<0.001^*∗*^	<0.001^*∗*^				

*6 weeks*							
*N*	38	39	38				
Mean (SD)	14.5 (6.3)	10.4 (4.1)	14.3 (5.8)	0.003^*∗*^	0.001^*∗*^	0.002^*∗*^	0.466
Decrease from baseline (SD)	10.3 (9.8)	9.3 (6.0)	8.7 (7.5)	0.471			
*P* value compared to baseline	<0.001^*∗*^	<0.001^*∗*^	<0.001^*∗*^				

*3 months*							
*N*	32	34	35				
Mean (SD)	13.3 (3.7)	11.6 (2.3)	13.1 (3.9)	0.197			
Decrease from baseline (SD)	10.9 (8.4)	8.0 (5.8)	10.2 (8.0)	0.196			
*P* value compared to baseline	<0.001^*∗*^	<0.001^*∗*^	<0.001^*∗*^				

*6 months*							
*N*	36	33	31				
Mean (SD)	12.7 (3.8)	10.4 (2.4)	13.2 (3.5)	<0.001^*∗*^	0.004^*∗*^	<0.001^*∗*^	0.121
Decrease from baseline (SD)	12.0 (9.0)	8.1 (4.5)	9.7 (7.3)	0.069			
*P* value compared to baseline	<0.001^*∗*^	<0.001^*∗*^	<0.001^*∗*^				

*1 year*							
*N*	34	29	24				
Mean (SD)	12.1 (3.8)	10.2 (2.8)	13.8 (4.0)	<0.001^*∗*^	0.009^*∗*^	<0.001^*∗*^	0.050^*∗*^
Decrease from baseline (SD)	12.9 (8.5)	8.3 (4.6)	7.4 (6.9)	0.010^*∗*^	0.020^*∗*^	0.177	0.002^*∗*^
*P* value compared to baseline	<0.001^*∗*^	<0.001^*∗*^	0.001^*∗*^				

*1.5 years*							
*N*	27	15	12				
Mean (SD)	10.8 (3.0)	9.4 (1.8)	11.7 (2.1)	0.034^*∗*^	0.059	0.005^*∗*^	0.074
Decrease from baseline (SD)	15.6 (7.7)	9.3 (5.0)	10.7 (8.9)	0.035^*∗*^	0.010^*∗*^	0.379	0.034^*∗*^
*P* value compared to baseline	<0.001^*∗*^	<0.001^*∗*^	0.004^*∗*^				

*2 years*							
*N*	12	7	7				
Mean (SD)	10.9 (1.9)	9.7 (2.7)	11.9 (2.3)	0.340			
Decrease from baseline (SD)	14.2 (7.4)	10.4 (4.1)	8.7 (6.3)	0.441			
*P* value compared to baseline	0.003^*∗*^	0.022^*∗*^	0.031^*∗*^				

^a^Preoperative and postoperative IOP outcomes data were analyzed using Kruskal-Wallis tests. Pairwise comparisons were conducted following significant Kruskal-Wallis test results using Dunn's test. IOP = intraocular pressure; mmHg = millimeters of mercury; AC = anterior chamber; *S* = sulcus; PP = pars plana; *N* = Number of eyes; SD = standard deviation.

**Table 4:** Medication burden outcomes data^a^.

	Medications (#)			*P* value			
	*AC*	*S*	*PP*	*All groups*	*AC* ∼ *S*	*S* ∼ *PP*	*AC* ∼ *PP*
*Preoperative*							
*N*	40	40	40				
Mean (SD)	4.0 (1.1)	4.2 (1.1)	4.1 (1.0)	0.827			

*1 day*							
*N*	40	40	39				
Mean (SD)	0 (0.0)	1.0 (1.8)	0.1 (0.6)	<0.001^*∗*^	<0.001^*∗*^	<0.001^*∗*^	0.243
Decrease from baseline (SD)	4.0 (1.1)	3.2 (2.0)	3.9 (1.2)	0.321			
*P* value compared to baseline	<0.001^*∗*^	<0.001^*∗*^	<0.001^*∗*^				

*2 weeks*							
*N*	38	37	39				
Mean (SD)	2.3 (2.1)	3.1 (1.7)	0.8 (1.2)	<0.001^*∗*^	0.039^*∗*^	<0.001^*∗*^	<0.001^*∗*^
Decrease from baseline (SD)	1.7 (2.1)	1.2 (1.5)	3.3 (1.4)	<0.001^*∗*^	0.166	<0.001^*∗*^	<0.001^*∗*^
*P* value compared to baseline	<0.001^*∗*^	<0.001^*∗*^	<0.001^*∗*^				

*6 weeks*							
*N*	38	39	38				
Mean (SD)	2.6 (1.8)	2.9 (1.4)	1.3 (1.4)	<0.001^*∗*^	0.240	<0.001^*∗*^	<0.001^*∗*^
Decrease from baseline (SD)	1.4 (2.0)	1.2 (1.2)	2.8 (1.5)	<0.001^*∗*^	0.447	<0.001^*∗*^	<0.001^*∗*^
*P* value compared to baseline	<0.001^*∗*^	<0.001^*∗*^	<0.001^*∗*^				

*3 months*							
*N*	32	34	35				
Mean (SD)	2.8 (1.4)	2.6 (1.3)	1.6 (1.4)	0.004^*∗*^	0.299	0.005^*∗*^	<0.001^*∗*^
Decrease from baseline (SD)	1.2 (1.6)	1.6 (1.0)	2.5 (1.3)	<0.001^*∗*^	0.252	0.002^*∗*^	<0.001^*∗*^
*P* value compared to baseline	<0.001^*∗*^	<0.001^*∗*^	<0.001^*∗*^				

*6 months*							
*N*	36	33	31				
Mean (SD)	2.8 (1.6)	2.7 (1.1)	1.6 (1.4)	0.002^*∗*^	0.400	0.002^*∗*^	<0.001^*∗*^
Decrease from baseline (SD)	1.2 (1.7)	1.5 (1.0)	2.4 (1.2)	0.002^*∗*^	0.352	0.002^*∗*^	<0.001^*∗*^
*P* value compared to baseline	<0.001^*∗*^	<0.001^*∗*^	<0.001^*∗*^				

*1 year*							
*N*	34	29	24				
Mean (SD)	2.9 (1.4)	2.7 (1.4)	1.5 (1.2)	<0.001^*∗*^	0.206	0.002^*∗*^	<0.001^*∗*^
Decrease from baseline (SD)	1.0 (1.5)	1.3 (1.2)	2.4 (1.3)	0.001^*∗*^	0.314	0.002^*∗*^	<0.001^*∗*^
*P* value compared to baseline	0.002^*∗*^	<0.001^*∗*^	<0.001^*∗*^				

*1.5 years*							
*N*	27	15	12				
Mean (SD)	3.0 (1.4)	2.8 (1.2)	1.7 (1.1)	0.017^*∗*^	0.567	0.019^*∗*^	0.002^*∗*^
Decrease from baseline (SD)	1.0 (1.5)	1.2 (0.8)	2.3 (1.1)	0.004^*∗*^	0.311	0.007^*∗*^	<0.001^*∗*^
*P* value compared to baseline	0.004^*∗*^	0.001^*∗*^	0.004^*∗*^				

*2 years*							
*N*	12	7	7				
Mean (SD)	2.9 (1.6)	2.6 (1.3)	1.9 (1.4)	0.330			
Decrease from baseline (SD)	1.3 (1.6)	1.1 (1.2)	2.1 (1.5)	0.404			
*P* value compared to baseline	0.024^*∗*^	0.066	0.036^*∗*^				

^a^Preoperative and postoperative medication burden outcomes data were analyzed using Kruskal-Wallis tests. Pairwise comparisons were conducted following significant Kruskal-Wallis test results using Dunn's test. AC = anterior chamber; *S* = sulcus; PP = pars plana; *N* = Number of eyes; SD = standard deviation.

**Table 5:** Visual acuity outcomes data^a^.

	Visual acuity (LogMAR)			*P* value			
	*AC*	*S*	*PP*	*All groups*	*AC* ∼ *S*	*S* ∼ *PP*	*AC* ∼ *PP*
*Preoperative*							
*N*	40	40	40				
Mean (SD)	0.35 (0.32)	0.29 (0.31)	0.89 (0.86)	<0.001^*∗*^	0.164	<0.001^*∗*^	<0.001^*∗*^

*1 day*							
*N*	40	40	39				
Mean (SD)	0.83 (0.74)	1.14 (0.91)	1.35 (0.79)	0.003^*∗*^	0.055	0.031^*∗*^	<0.001^*∗*^
Decrease from baseline (SD)	−0.48 (0.55)	−0.69 (1.06)	−0.45 (0.84)	0.798			
*P* value compared to baseline	<0.001^*∗*^	<0.001^*∗*^	0.010^*∗*^				

*2 weeks*							
*N*	38	37	39				
Mean (SD)	0.60 (0.78)	0.52 (0.45)	1.05 (0.81)	<0.001^*∗*^	0.324	<0.001^*∗*^	<0.001^*∗*^
Decrease from baseline (SD)	−0.26 (0.67)	−0.11 (0.57)	−0.20 (0.85)	0.923			
*P* value compared to baseline	0.009^*∗*^	<0.001^*∗*^	0.147				

*6 weeks*							
*N*	38	39	38				
Mean (SD)	0.46 (0.43)	0.29 (0.23)	0.83 (0.66)	<0.001^*∗*^	0.041^*∗*^	<0.001^*∗*^	0.002^*∗*^
Decrease from baseline (SD)	−0.08 (0.37)	−0.01 (0.32)	0.11 (0.66)	0.526			
*P* value compared to baseline	0.269	0.586	0.933				

*3 months*							
*N*	32	34	35				
Mean (SD)	0.47 (0.56)	0.27 (0.21)	0.79 (0.73)	<0.001^*∗*^	0.036^*∗*^	<0.001^*∗*^	0.009^*∗*^
Decrease from baseline (SD)	−0.03 (0.31)	0.02 (0.31)	0.13 (0.73)	0.595			
*P* value compared to baseline	0.524	0.792	0.501				

*6 months*							
*N*	36	33	31				
Mean (SD)	0.47 (0.67)	0.25 (0.23)	0.90 (0.73)	<0.001^*∗*^	0.051	<0.001^*∗*^	<0.001^*∗*^
Decrease from baseline (SD)	−0.03 (0.42)	−0.02 (0.34)	0.16 (0.89)	0.515			
*P* value compared to baseline	0.980	0.595	0.408				

*1 year*							
*N*	34	29	24				
Mean (SD)	0.37 (0.54)	0.25 (0.21)	0.86 (0.75)	<0.001^*∗*^	0.297	<0.001^*∗*^	<0.001^*∗*^
Decrease from baseline (SD)	0.00 (0.46)	0.03 (0.32)	0.14 (0.94)	0.832			
*P* value compared to baseline	0.531	0.457	0.825				

*1.5 years*							
*N*	27	15	12				
Mean (SD)	0.48 (0.60)	0.37 (0.29)	0.70 (0.86)	0.713			
Decrease from baseline (SD)	−0.04 (0.27)	−0.07 (0.19)	0.47 (0.97)	0.135			
*P* value compared to baseline	0.365	0.126	0.155				

*2 years*							
*N*	12	7	7				
Mean (SD)	0.57 (0.86)	0.50 (0.24)	0.68 (0.62)	0.525			
Decrease from baseline (SD)	−0.11 (0.25)	−0.06 (0.18)	0.39 (1.28)	0.962			
*P* value compared to baseline	0.086	0.529	0.787				

^a^Preoperative and postoperative visual acuity outcomes data were analyzed using Kruskal-Wallis tests. Pairwise comparisons were conducted following significant Kruskal-Wallis test results using Dunn's test. LogMAR = logarithm of minimum angle of resolution; AC = anterior chamber; *S* = sulcus; PP = pars plana; *N* = Number of eyes; SD = standard deviation.

**Table 6:** Life table.

	Cumulative success (%) ± SE (95% confidence interval)		
	IOP reduction ≥20% with 5<IOP ≤21 mmHg		
	*AC*	*S*	*PP*
3 months	100.0 ± 0.0	100.0 ± 0.0	100.0 ± 0.0
	(100.0, 100.0)	(100.0, 100.0)	(100.0, 100.0)

*n*	36	30	35
6 months	97.2 ± 2.7	96.6 ± 3.4	96.6 ± 3.4
	(92.0, 100.0)	(90.1, 100.0)	(90.1, 100.0)

*n*	35	27	26
1 year	94.2 ± 4.0	87.8 ± 6.8	87.5 ± 6.9
	(86.7, 100.0)	(75.5, 100.0)	(75.1, 100.0)

*n*	31	16	17
1.5 years	94.2 ± 4.0	87.8 ± 6.8	74.9 ± 10.3
	(86.7, 100.0)	(75.5, 100.0)	(57.2, 98.1)

*n*	20	5	7
2 years	94.2 ± 4.0	87.8 ± 6.8	74.9 ± 10.3
	(86.7, 100.0)	(75.5, 100.0)	(57.2, 98.1)

*n*	3	1	4
*P value* ^a^	0.2		

SE = standard error; IOP = intraocular pressure; AC = anterior chamber; *S* = sulcus; PP = pars plana; *n* = number of eyes. ^a^Success rates across the 3 treatment groups were compared using a log rank test.

**Table 7:** Hazard ratios from univariate Cox proportional hazard models using demographic and preoperative data^a^.

Univariate model parameters	IOP reduction ≥20% with 5<IOP ≤21 mmHg	
	HR (95% CI)	*P* value
Age	0.994 (0.956–1.035)	0.8
Sex (female)		0.4
Male	1.590 (0.551–4.588)	

Glaucoma stage (indeterminate)		0.5
Mild	<0.001 (0–Inf)	
Moderate	0.900 (0.100–8.090)	
Severe	0.407 (0.052–3.217)	

Race^b^		-
Family history of glaucoma (no)		0.2
Yes	0.504 (0.158–1.610)	—
Diagnosis^b^	—	

History of CPC (no)		0.1
Yes	2.492 (0.834–7.443)	

Type of glaucoma implant (AGI)		0.4
BGI	0.658 (0.228–1.896)	

Preoperative IOP	0.594 (0.489–0.721)	<0.001^*∗*^
Preoperative medication burden	0.948 (0.572–1.57)	0.8
Tube location (AC)		0.2
Sulcus	3.727 (0.721–19.27)	—
Pars plana	4.369 (0.9029–21.14)	

IOP = intraocular pressure; mmHg = millimeters of mercury; HR = hazard ratio; CI = confidence interval; Inf = infinity; CPC = cyclophotocoagulation; AGI = Ahmed glaucoma implant; BGI = Baerveldt glaucoma implant; AC = anterior chamber. ^a^Hazard ratios and significance were calculated using univariate Cox proportional hazard models. ^b^The model was not able to estimate a hazard ratio for this variable due to lack of sufficient sample size in each of its categories.

**Table 8:** Complication rates^a^.

	*N*	*n* (%)			
		AC inflammation	Hypotony	Corneal edema	Cystoid macular edema
*Total*					
All groups	120	64 (53.3)	12 (10.0)	46 (38.3)	18 (15.0)
AC	40	20 (50.0)	6 (15.0)	11 (27.5)	5 (12.5)
*S*	40	33 (82.5)	5 (12.5)	24 (60.0)	6 (15.0)
PP	40	17 (42.5)	1 (2.5)	12 (30.0)	8 (20.0)
*P* value		<0.001^*∗*^	0.149	0.005^*∗*^	0.741

*Early* ^b^					
All groups	120	64 (53.3)	12 (10.0)	46 (38.3)	18 (15.0)
AC	40	20 (50.0)	6 (15.0)	11 (27.5)	5 (12.5)
S	40	33 (82.5)	5 (12.5)	24 (60.0)	6 (15.0)
PP	40	16 (40.0)	1 (2.5)	12 (30.0)	8 (20.0)
*P* value		<0.001^*∗*^	0.149	0.005^*∗*^	0.741

*Late* ^c^					
All groups	100	2 (2.0)	0 (0.0)	6 (6.0)	14 (14.0)
AC	36	1 (2.8)	0 (0.0)	4 (11.1)	7 (19.4)
S	33	0 (0.0)	0 (0.0)	0 (0.0)	4 (12.1)
PP	31	1 (3.2)	0 (0.0)	4 (12.9)	7 (22.6)
*P* value		0.760	1.000	0.091	0.549

*N* = total number of patients at specific follow-up time; *n* = number of patients; AC = anterior chamber; *S* = sulcus; PP = pars plana. ^a^Complication rates were compared across the three treatment groups using a Fisher test. ^b^Complications present up to 3 months postoperatively. ^c^Complications present after 3 months postoperatively.

**Table 9:** Causes for >1 line decrease in VA.

	*n* (%)			
	Total	AC	*S*	PP
CME	3	1 (2.5)	1 (2.5)	1 (2.5)
PCO	2	0 (0.0)	2 (5.0)	0 (0.0)
Macular degeneration	2	1 (2.5)	0 (0.0)	1 (2.5)
Corneal graft failing	2	0 (0.0)	0 (0.0)	2 (5.0)
Blau syndrome	1	0 (0.0)	0 (0.0)	1 (2.5)
Corneal edema	1	0 (0.0)	0 (0.0)	1 (2.5)
Postoperative hemorrhagic choroidal detachment	1	0 (0.0)	0 (0.0)	1 (2.5)
Wet AMD	1	1 (2.5)	0 (0.0)	0 (0.0)
RVO	1	1 (2.5)	0 (0.0)	0 (0.0)
Vitreous hemorrhage from open globe	1	1 (2.5)	0 (0.0)	0 (0.0)
Poorly controlled IOP	1	1 (2.5)	0 (0.0)	0 (0.0)
*Total*		6	3	7
*P* value^a^	0.3916			

VA = visual acuity; *n* = number of eyes; AC = anterior chamber; *S* = sulcus; PP = pars plana; CME = cystoid macular edema; PCO = posterior capsular opacification; AMD = age-related macular degeneration; RVO = retinal vein occlusion; IOP = intraocular pressure. ^a^The proportion of eyes with VA loss across the 3 treatment groups was compared using a three-sample test for equality of proportions.

## Data Availability

The data used to support the findings of the study are available upon request (sandy.samuel96@gmail.com).
